# Rationale for Mass Masking in Controlling the COVID-19 Pandemic

**DOI:** 10.3389/fpubh.2021.665708

**Published:** 2021-08-24

**Authors:** Shing Yau Tam, Victor C. W. Tam, Helen K. W. Law, May Ling Khaw, Shara W. Y. Lee

**Affiliations:** ^1^Department of Health Technology and Informatics, Faculty of Health and Social Sciences, The Hong Kong Polytechnic University, Kowloon, China; ^2^Tasmanian School of Medicine, University of Tasmania, Hobart, TAS, Australia

**Keywords:** COVID-19, pandemic, public health, public health policy, face mask, infection control, mass masking

## Abstract

The rapid spread of the coronavirus disease 2019 (COVID-19) into a global pandemic caught the world unprepared. Previously effective measures for containing disease outbreaks were overwhelmed, necessitating strict controls such as lockdowns or curfews. Among the disease control interventions, community mass masking was one of the highly controversial issues with differing opinions on its indications or effectiveness from different health authorities around the world. Regions where community mass masking was timely introduced were associated with lower transmission rates, and more effective disease control. In this article, we discuss the evidence on the effectiveness, and rationale for community mass masking to prevent the COVID-19 transmission. Areas for further research to define the role of mass masking in light of the COVID-19 pandemic will be suggested. This would help policy makers in formulating mass masking policies.

## Introduction

The coronavirus disease 2019 (COVID-19) is the result of infection by the novel severe acute respiratory syndrome coronavirus 2 (SARS-CoV2), making this the third coronavirus to have crossed species and cause severe disease in humans. In the absence of effective vaccine distribution or specific antiviral agents, the only means of limiting transmission is through physical interventions such as mask wearing, physical distancing, and isolation of infectious individuals. Although the routes of transmission via respiratory droplets or aerosols and direct contact are similar to other coronaviruses, COVID-19 has defeated the control measures of many high-income nations with well-funded health infrastructures.

There are several characteristics of the COVID-19 infection that has enabled it to spread efficiently and rapidly developed into a global pandemic. COVID-19 has a wide spectrum of presentations that range from asymptomatic or having only mild symptoms, to systemic illness with multiorgan involvement (1–4). Severity of illness is generally increased in those at a more advanced age, while the younger carriers have only mild symptoms and are consequently able to spread the virus undetected. Another feature of the COVID-19 infection is the long presymptomatic phase, with a median incubation period of 5–6 days and can be as long as 19 days (5–8). Transmission can occur during the latter part of the presymptomatic phase, and 48–62% of infections were attributed to presymptomatic carriers (9). Moreover, these presymptomatic carriers were able to pass undetected through temperature screening, a previously dependable process for disease control at border checkpoints, and therefore facilitating the rapid spread globally.

As one of the key factors for limiting the transmissibility of COVID-19, community mass masking was one of the public health measures used to mitigate its rapid spread. Initially, this practice is not universally adopted, which may account for poorer infection control outcomes for some regions (10, 11). The issue of community mass masking at the time of the emergence of COVID-19 has been highly controversial, with conflicting recommendations issued by health authorities around the world. In an earlier guideline during the spread of the pandemic from East to West, the World Health Organization (WHO) conservatively recommended against community mass masking with medical masks due to the lack of concrete evidence, while stressed on reserving the scarce resources for the healthcare workers (12). Although explicable, it seemed to reduce the chance to limit the substantial spreading of the disease in the early stage. This early recommendation was also accompanied by a speculative suggestion of risk compensation with mass masking, leading to the reduced attention in other effective protective behaviors such as performing hand hygiene (6, 12). On the contrary, Mantzari et al. (13) systematically reviewed previous studies on wearing mask with hand hygiene in community settings for controlling respiratory infections. They concluded that wearing masks did not reduce the frequency of hand sanitizing while two studies reported higher hand washing rates in the groups with mask wearing.

Notably, the recommendations on mass masking were made based on the evidence from studies on influenza outbreaks, which were not necessarily applicable to the present coronavirus. Given the little amount that was known about the COVID-19 disease and its modes of transmission, many health authorities in Asia (e.g., China, Hong Kong, Singapore etc.) exercised caution by implementing community masking recommendations (12). Subsequent recommendations based on the experience of successful control of early COVID-19 outbreaks in China also advocated for mass masking (14). Generally, Asian societies appear more inclined to wear masks voluntarily when unwell, or for protection during disease outbreaks, given the previous close encounters with epidemics such as the severe acute respiratory syndrome (SARS) in 2003 that predominantly affected Asia. Consequently, compliance with community mass masking recommendations has been high in these regions. In a global survey conducted across 15 countries during the pandemic (April 9–12, 2020) (15), countries with significant proportions of mask wearers included Vietnam (91%), China (83%), Italy (81%), Japan (77%), and India (76%). By comparison, this was lower in Western countries including United Kingdom (16%), Germany (20%), Australia (21%), Canada (28%), and France (34%).

Evidence from epidemiological data show that with a timely introduction of community mass masking in conjunction with infection prevention and control strategies were more effective in containing the COVID-19 transmission (10, 16, 17). Escalation of stricter controls such as lockdowns or curfews were subsequently not necessary (10), as exemplified in Taiwan (18). The aim of this article is to discuss the current evidence on the effectiveness, and rationale for community mass masking in preventing COVID-19 transmission. Areas for further research in order to define the role of mass masking in light of the COVID-19 pandemic will be discussed.

## Rationale for Mass Masking

Before examining the role of masks in mitigating the COVID-19 transmission, its function and basic mechanism of protection should first be considered. A mask provides a semipermeable barrier to limit the passage of particles or substance through it. The relative size of the particle to that of the pores is important. Thus, the purpose of use should be considered before a type of mask is selected. Medically speaking, surgical masks were originally used as source control to prevent contamination by droplets or aerosols containing microbes from the nose and throat of theater staff to the patient's site of operation. The use of surgical masks nowadays also protect staff from potential biohazards encountered in the operating theater, where N95 respirators are also used. Surgical masks and N95 respirators are made of high filtration materials with efficacy of ≥95% (0.3 μm). The protective effect of a medical mask is rated based on its filtration efficacy and the ability to provide an airtight fit to the face. When loose fitting and therefore permitting the passage of air around the edges, there is a drop in overall rated filtration efficacy to <70%, according to the US National (N10SH) N95 test (19). For this reason, the surgical mask is not classified as personal protective equipment (PPE) (20), whereas a well-fitted respirator with a N95 rating fulfills the designation of PPE due to its airtight design, and has a filtration efficacy of ≥95% (19).

When coughing or sneezing, aerosols, and droplet particles are exhaled, which range from 0.1 to 1,000 μm in size (11). Aerosols are particles sized <5 μm and they remain airborne for a longer period. The spread of these particles is determined by numerous factors including size, ambient temperature, air movement, and humidity. Droplets may partially evaporate, which cause them to gain more buoyancy and remain airborne for longer to spread over a greater area (11). The smaller submicron (<1 μm) aerosol particles are considered to be more dangerous as they can be inhaled and lodged deeper into the lung parenchyma, leading to more severe infections due to interference with gas exchange (20, 21).

## Discussion

Recent studies (21, 22) have reported that the COVID-19 virus detected in both droplets and aerosols can be propelled further than the recommended one meter of social distancing by the WHO (13, 23), or six feet recommendation by the US Centre for Disease Control and Prevention (CDC) (24).

The WHO previously recommended against wearing masks in the community due to a lack of evidence on its effectiveness. However, the evidence underlying this recommendation was drawn from 10 randomized studies on transmission of the influenza, and other human coronaviruses in the community (6). These findings could be not applicable to the transmission of a novel coronavirus which spreads more surreptitiously than influenza due to the presymptomatic carriers and those with mild symptoms that evaded detection. The basic reproductive rate of COVID-19 was estimated to be between 2.2 and 2.7 (23, 25), while the rate of the 2009 influenza H1N1 pandemic was estimated to be 1.7 (26). Subsequently in June 2020, the WHO guideline was revised to suggest mass masking as part of the comprehensive strategy if there is limited capacity of measures in light of ongoing transmission by asymptomatic carriers in the community settings, and the potential difficulties in maintaining physical distancing and containment measures (27). The importance of mass masking in mitigating COVID-19 pandemic is summarized in Table 1.

**Table 1:** Summary of the evidence for mass masking in mitigating COVID-19 pandemic.

**Evidence**	**Theme(s)**	**References**
- Non-pharmaceutical interventions, including population behavioral changes in masking and social distancing, were associated with reduced transmission of COVID-19 and influenza.	A	(10)
- Community mask use by well people was suggested to offer protection to healthy individuals especially in high transmission settings.	A	(16)
- Presymptomatic transmission contributed to a significant proportion of the COVID-19 transmission. - Control strategies, such as community masking, should be adjusted to tackle presymptomatic transmission.	B	(17)
- Presymptomatic patients with mask-wearing showed significantly lower transmission to close-contact persons.	A, B	(28)
- Surgical mask partition reduced transmission of COVID-19 in a hamster model. - Reduction in transmission between the sick and the healthy groups was more significant when the surgical mask partition was placed in the sick hamsters' compartment compared to placing it in the healthy hamsters' compartment.	A	(29)
- Surgical face masks reduced viral RNA in respiratory droplets and aerosols from exhaled breath and coughs.	A, C	(30)
- Use of cloth face coverings by both symptomatic patients and healthy individual might reduce the risk of transmission.	A	(31)
- The relatively low incidence of COVID-19 in Hong Kong might be contributed by the high compliance of mass masking. - Transmission clusters in ‘mask-off' settings were more prevalent than that in ‘mask-on' settings.	A	(32)
- Airborne transmission was showed to be highly virulent and represented the dominant route to spread the disease in confined environment. - Social distancing alone might be insufficient in protecting the public. - Mandatory face-covering appeared to reduce transmission.	A, C	(33)
- Mask non-wearing rate was shown to be a strong predictor of the numbers of death of COVID-19 pandemic across 22 countries.	D	(34)
- Mathematical modeling evaluated that mass masking could reduce total infections and deaths in COVID-19 pandemic. Also, it could delay the peak time of the epidemic.	D, E	(35)

*A, reduction in transmissibility; B, reduction in presymptomatic spread; C, reduction in droplet and aerosol transmission; D, reduction in death rate; E, delay in peak time of the epidemic*.

Reports of transmission by asymptomatic carriers with a high viral load at the initial stage of infection emerged as early as February 2020 (5, 36), with the peak viral load occurring during symptom onset (37, 38). A recent meta-analysis of 9 published reports concluded that 15% of COVID-19 infections were asymptomatic (39), and the incubation period was reported to be 5–6 days, but can be as long as 19 days (5–8). Virus shedding in the upper respiratory tract is high among presymptomatic patients (40), while those with asymptomatic influenza usually have a lower viral load in the upper than the lower respiratory tract (41). This is the new challenge posed by the COVID-19 virus that distinguishes it from most other infectious diseases. Asymptomatic carriers were reported to play a significant role in the community spread. Li et al. (42) estimated that 86% (95% CI: 82–90%) of all infections were undocumented before travel restrictions were implemented in China. Although the transmission rate of undocumented infections was lower than the documented ones, it is estimated to be responsible for 48–62% of the COVID-19 infections (1). A recent study conducted by Hong et al. (28) reported a significantly higher incidence of COVID-19 among individuals who had close contact with presymptomatic patients without wearing masks (19.0 vs. 8.1%, *p* < 0.001).

Aerosol particles are smaller, and depending on the physical conditions such as humidity, temperature or wind, can linger in the air for considerably longer compared to droplets (20), thus enabling the virus to spread more efficiently despite physical distancing. The decay rates ranged from 0.4 to 2.27% per minute and the half-lives 30–177 min under different conditions with lower relative humidity as a factor for longer half-lives (43). Ultimately, the likelihood of infection with COVID-19 is highly dependent on the contaminating viral dose during transmission (44, 45). The rationale for mask wearing is firstly recognized as a source control to minimize the amount of virus exhaled by carriers to the atmosphere. With masking, both the amount and velocity of droplets or aerosols expelled in a breath will be smaller, thus reducing the area of spread. Secondly, the mask will protect healthy individuals from exposure to droplets or aerosols (29, 46).

Leung et al. (30) prospectively evaluated the transmission of coronavirus, influenza virus and rhinovirus in droplets and aerosols of the exhaled breath of patients with acute respiratory illnesses. They found that while only some carriers exhaled a detectable viral load, wearing a surgical mask was effective in blocking the transmission of both droplets and aerosols completely. Bae et al. (47) investigated the effectiveness of cotton and surgical masks for preventing virus shedding during coughing by COVID-19 patients. In contrast, they found that neither surgical nor cotton masks could filter the virus effectively, which was attributed to the production of aerosols when coughing. These findings suggest that although the surgical mask is adequate as a source control for asymptomatic carriers in the community, it could be less effective for those who are actively coughing. Symptomatic individuals will need testing and isolation; for source control, the N95 respirator may be required.

Meanwhile, the revised WHO guideline recommended the use of non-medical cloth or homemade masks in the community in order to preserve the stock of medical masks for healthcare workers, and recommended these as a means of source control in community settings but not for prevention (27). The guideline also suggested that wearing cloth masks on public transport, frequent hand hygiene and physical distancing should always be adopted together with cloth masks. Following the revised guideline from the WHO in June 2020 on community mass masking, many countries have adopted this recommendation. However, cloth masks may have varying filtering efficiencies, which are generally lower than that of the medical masks (48, 49). There is a lack of solid evidence to support the effectiveness of using cloth mask as a mean of source control to the asymptomatic carriers. A report conducted by Hendrix et al. (31) described 139 clients with cloth face coverings were not infected with COVID-19 after contact with two symptomatic hair stylists who also wore face coverings. The hair stylists were eventually tested positive. Yet, one laboratory study reported inferior filtering performance of general masks compared to medical masks (50). Paradoxically, the only clinical outcome study by Zhang et al. (33) reported a higher rate of respiratory illness among hospital staff wearing a cloth mask compared to not wearing a mask for protection at work. The efficacy of using cloth mask in the community setting has never been evaluated in an outcome study for COVID-19.

With the easing of lockdown in many areas, it can be seen that social distancing on its own is inadequate, and the infection rates have increased considerably. Epidemiological data from Asian regions or countries such as China, Hong Kong, Japan, Macau, Singapore, South Korea (14), Taiwan (15) where universal masking was enforced or recommended showed effective control of COVID-19 transmission, and implementation of lockdowns were limited. A number of infections occurred in bars or restaurants where masks were often removed, which emphasizes the importance of this measure (32). A recent study analyzed the trend and mitigation measures in Wuhan, Italy, and New York City from 23 January to 9 May 2020. The findings concluded that the single determinant shaping the pandemic trend was the imposition of mandatory face covering. Surprisingly, other measures such as social distancing failed to suppress transmission (33). Miyazawa et al. (34) reported a strong inverse relationship between the masking rate to the mortality rate from COVID-19; with a predictive power of 69% based on an analysis of data from 22 countries including 13 western and 9 Asian countries in March 2020. Worby and Chang (35) employed mathematical modeling to evaluate the epidemiological impact of mass masking among general population by considering resource limitations, supply and demand dynamics. They concluded that face masks can reduce total infections and deaths in COVID-19 pandemic even if the protective effect of face masks is limited. In addition, the peak time of the epidemic could be delayed. MacIntyre et al. (16) concluded in a systematic review that wearing a mask in the community was effective for both protection in crowded areas, and as a source control for COVID-19 transmission. They added that mass masking would be more effective if implemented early during an outbreak, which was the case for many Asian countries. Cowling et al. (10) supported this notion by suggesting that non-pharmaceutical interventions including wearing masks among citizens of Hong Kong may contribute to the suppression of local transmission of COVID-19.

A recent study conducted in Hong Kong, a population that is very compliant to community mass masking (94.8%), reported that the majority of masked individuals (83.7%) wore surgical masks. From the online questionnaire respondents, 76.3% reused their masks (51). The low infection rate at the initial stage of outbreak may imply that the reuse of surgical masks in community settings is not as harmful as anticipated, and that the benefits of masking outweigh the risks of reusing masks. A cross-sectional study in Brazil also showed similar percentage of mask reuse (71.1%) (52). However, it is notable that the efficacy or performance of reused masks is not evaluated. There was one Canadian study evaluating on the reuse of N95 respirators by autoclaving (53). Findings suggested that reusing N95 respirators was feasible in hospitals with successful reuse rate ranging from 48.8% to 79.6% in 12 sterilization cycles. The issue of early global medical mask shortage could be addressed by rationing, such as in South Korea (52) and Taiwan (53), or by the provision of free, high efficacy reusable masks in Hong Kong (54) and Singapore (54). Recently, the worldwide supply shortage of medical mask has eased considerably, and this is no longer an issue impeding the implementation of mass masking policy for transmission control in many developed countries.

## Further Research and Directions

The COVID-19 pandemic has exposed a number of weaknesses in the readiness and competence of many health authorities around the world in handling a novel infectious disease. The Chinese government resorted to the use of draconian measures to bring control to the transmission, yet many countries remained complacent and underprepared for its malignant spread. Health authorities will benefit from reviewing their capabilities in order to formulate a better epidemic response plan. Although mass masking was recommended based on lessons gained from the experience in China, this was not closely followed in many countries possibly due to the WHO's earlier guidelines against community masking. Reports of presymptomatic carriers and also transmission via aerosol route were largely disregarded. Prompt and continuous re-evaluation and adoption of newly evident recommendations by health authorities would be essential to savagely control the outbreak of the novel virus.

Future research should be directed to address these deficits. The effectiveness of repeated used cloth masks in limiting the spread of respiratory diseases, which address the shortage of disposable surgical masks. The filtration efficiency and safety test using a standardized protocol worldwide should be adopted to facilitate interpretation and comparison. The reusable copper mask that incorporates antimicrobial properties to its filter is an example of innovation, spurred by the mask shortage in Hong Kong (55). The mask complies with the American Society of Testing and Materials (ASTM) F2100 Level 1 standard and can be used in community settings. While Gilbert et al. (56) proposed an inexpensive ultraviolet system for filtering facepiece respirator decontamination that may ease the respirator demand. Efforts should be directed to the design of masks made to improve protection against aerosols that can be easily mass-produced, and safely reused by appropriate decontamination devices.

## Conclusions

We have summarized and discussed the evidence on community mass masking as a public health measure for controlling the COVID-19 transmission. Regions where mass masking was timely introduced were effective with disease control (10), potentially leading to fewer cases of mortality (57). As a source control, the surgical mask is effective, but only if carriers are asymptomatic and do not cough. With easing of lockdowns, the role of mass masking becomes more vital and remains an essential measure for controlling transmission. Lastly, the most important lesson to learn from the COVID-19 pandemic is to focus on better preparation, communication, and international cooperation to effectively limit the spread of emerging infectious diseases in the future.

## Data Availability Statement

The original contributions presented in the study are included in the article/supplementary material, further inquiries can be directed to the corresponding author/s.

## Author Contributions

ST and VT performed the literature search. ST, VT, MK, and SL collected information and drafted the manuscript. SL supervised the study and edited the manuscript. ST, VT, HL, and MK edited the manuscript. All authors contributed to the article and approved the submitted version.

## Conflict of Interest

The authors declare that the research was conducted in the absence of any commercial or financial relationships that could be construed as a potential conflict of interest.

## Publisher's Note

All claims expressed in this article are solely those of the authors and do not necessarily represent those of their affiliated organizations, or those of the publisher, the editors and the reviewers. Any product that may be evaluated in this article, or claim that may be made by its manufacturer, is not guaranteed or endorsed by the publisher.
